# Looking at Self-Disorders through the Minnesota Multiphasic Personality Inventory (MMPI): An empirical exploration of the MMPI-derived Self-Disorder Scale

**DOI:** 10.1192/j.eurpsy.2021.2027

**Published:** 2021-08-13

**Authors:** E. Monducci, G. Colafrancesco, G. Perrotti, G. De Vita, L. Quadrana, M. Ferrara

**Affiliations:** 1 Department Of Human Neurosciences, University of Rome Sapienza, Rome, Italy; 2 Department Of Human Neurosciences, section Of Child And Adolescent Neuropsychiatry, University of Rome Sapienza, Rome, Italy

**Keywords:** Minnesota Multiphasic Personality Inventory, Self-Disorders, adolescence, schizophrénia

## Abstract

**Introduction:**

Trait-like anomalies of subjective experience have been empirically identified as schizophrenia-specific markers of vulnerability in several clinical and genetic high-risk populations. Recently, Parnas and colleagues have identified and preliminarily explored a composite score (i.e. Self-Disorder Scale, SDO) within the Minnesota Multiphasic Personality Inventory (MMPI) that approximates such construct). SDO differs from the MMPI psychoticism scale, and includes presents items very similar to Self Disorder investigated by EASE (Examination of Anomalous Self-experience).

**Objectives:**

This study is a confirmatory analysis of the correspondence of Self-Disorder Scale (SDO) of the MMPI with some items of EASE, in a population of adolescents. These items are present in psychotic and in at risk mental state subjects.

**Methods:**

We administered MMPI and EASE to 34 help seeker adolescent patients and correlate all dimensions of MMPI with EASE total score and its domains.

**Results:**

MMPI SDO scores significantly correlated with schizophrenia-spectrum diagnosis and high-risk mental states.

**Conclusions:**

SDO is an MMPI analogous of Self Disorders and can be used as a useful screener to detect patients at potential risk for schizophrenia spectrum disorders, that could be further explored with the EASE.

**Disclosure:**

No significant relationships.

